# The effect of psychological stress on masticatory muscle electromyography—a scoping review

**DOI:** 10.22514/jofph.2026.047

**Published:** 2026-07-12

**Authors:** Alexander Weden, Michail Koutris, Hedwig van der Meer

**Affiliations:** ^1^Department of Maxillofacial & Physiotherapy, Queens Medical Centre, Nottingham University Hospitals, NG7 2UH Nottingham, UK; ^2^Association of Chartered Physiotherapists in Temporomandibular Disorders (ACPTMD), BD19 9DN Cleckheaton, UK; ^3^Department of Orofacial Pain and Dysfunction, Academic Centre for Dentistry Amsterdam (ACTA), University of Amsterdam and Vrije Universiteit (VU) University Amsterdam, 1081LA Amsterdam, The Netherlands; ^4^SOMT University of Physiotherapy, 3821BN Amersfoort, The Netherlands

**Keywords:** Temporomandibular disorders, Psychological stress, Electromyography, Scoping review

## Abstract

Temporomandibular disorders (TMDs) are the second most common reason for orofacial pain with myalgia being the most prevalent diagnosis. Overuse of the masticatory muscles and the presence of stress are both risk factors for myalgia. The extent to which these risk factors interact remains unclear, including whether psychological stress contributes to increased masticatory muscle activity. The purpose of this scoping review was to identify, examine, and map the available literature exploring the relationship between psychological stress and masticatory muscle electromyography (EMG). Pre-clinical and clinical studies that studied adults with or without TMD who were exposed to psychological stress and had masticatory muscle electromyography recordings were included. The electronic databases CINAHL, EMBASE, Medline, and PsycINFO were searched in March 2025. Using a pre-defined data extraction sheet, basic study information and specific results regarding the research question were extracted. These were analysed and the results were described based on the type of study (pre-clinical *vs.* clinical), as well as the type of stress (experimental *vs.* clinical). Following screening, 38 studies (published between 1971 and 2025) were included: 29 clinical experimental studies, 3 cross-sectional studies and 6 pre-clinical studies. All pre-clinical and cross-sectional studies demonstrated that psychological stress increased masticatory muscle EMG. Among the clinical studies, 25 of the 29 studies reported that psychological stress increased masticatory muscle EMG. Psychological stress consistently increased masticatory muscle EMG in both human and animal models, although some caution is warranted given the paucity of manipulation checks in the included studies. Putative biological mechanisms are discussed herein. A trend towards greater EMG amplitude in response to stress in patients with TMD compared to healthy controls was demonstrated. Given the high prevalence of psychological stress in Western populations, these findings argue that the presence of psychological stress should be consistently evaluated when assessing patients with TMD.

## 1. Introduction

Temporomandibular disorders (TMDs) are the second most common reason for orofacial pain after an odontogenic source. TMD is an umbrella term that refers to a variety of painful and dysfunctional symptoms of the masticatory system [1]. The most common taxonomy used to describe and classify the variety of TMD subtypes is the Diagnostic Criteria for Temporomandibular Disorders (DC/TMD) [2]. The most common type of DC/TMD diagnosis is myalgia of the masticatory muscles [1]. The aetiology of myalgia appears to be multifactorial and complex in nature and is often a combination of genetic predisposition, muscle overuse, the presence of psychosocial factors, and/or changes in central pain processing mechanisms [3, 4]. Historic orthognathic paradigms used to explain the aetiology of TMDs are now widely seen as misrepresentative, and viewing TMDs through the lens of the biopsychosocial model has become the dominant means by which TMDs should be conceptualised [5].

Masticatory muscle overloading, occurring during activities such as bruxism and oral parafunctions, can be a risk factor for developing myalgia as well as a prognostic factor for the transformation of acute myalgia into chronic myalgia [6]. Interestingly, patients with myalgia present with elevated masticatory muscle electromyography (EMG) both at rest and during oromotor function [7]. The co-presence of masticatory myalgia and elevated masticatory muscle EMG is widely acknowledged; however, the causal relationship remains elusive. Masticatory myalgia may precipitate elevated masticatory muscle EMG, but if elevated masticatory muscle tone (and thereby EMG) leads to myalgia, then elucidating what precipitates this change in muscle function would be critical in the search to prevent the condition and/or treat it “at its source”.

Another important risk factor and prognostic factor for (chronic) TMD is the presence of negative affective states such as anxiety, depression, and psychological stress [8]. These psychosocial factors have been consistently demonstrated to be highly comorbid with masticatory muscle pain [9]. Acute and chronic stress have even been shown to affect multiple biological processes down to the gene expression level of pathways implicated in pain amplification [10]. Similarly, chronic stress has a well-known deleterious effect on multiple biological systems within our body (referred to as allostatic load) [11].

The effect of stress on muscle function has been studied for several years [12], however, comprehensive reviews of the data remain sparse. Given the high prevalence of psychological stress in the general population and its accepted role in contributing to the development of TMDs, it is no surprise that primary studies have been undertaken to elucidate the relationship between stress and TMDs. Recent studies even show that the presence of stress and the patient’s beliefs about masticatory muscle overuse both have an impact on the presence of TMD [13]. However, in these studies, masticatory muscle overuse was often based on questionnaires rather than EMG data. Studies have been undertaken to explore psychological stress, masticatory myalgia, and masticatory muscle function via EMG, but to our knowledge, a comprehensive review collating and evaluating the data remains absent.

Scoping reviews have become a popular means of investigating and providing an overview of available literature on a topic [14]. Before a systematic review is undertaken, scoping reviews provide a broad and comprehensive insight into the research landscape around a topic, thereby providing better-focused questions for a systematic review [15]. To date, no scoping review looking at the relationship between psychological stress and masticatory muscle EMG exists, nor is there one underway registered on salient databases such as the International Prospective Register of Systematic Reviews database (PROSPERO) or the Open Science Framework. In lieu of this, a scoping review would be of value in identifying and collating the available evidence exploring the potential relationship between psychological stress and masticatory muscle function as measured by EMG. One review article looking at the relationship between psychological stress and the physical aspects of TMD included two studies looking at the effect of psychological stress on masticatory muscle function [16], whilst more studies may exist that are relevant to our research question.

The purpose of this scoping review is to identify, examine and map the available literature exploring the relationship between psychological stress and masticatory muscle EMG. Commensurate with the opportunity scoping reviews afford to map the breadth of the literature on a topic, this scoping review used temporally deep and broad inclusion criteria to ensure a comprehensive search of the literature base was undertaken. The focus of the review was on the influence of psychological stress (as opposed to physical stress) and its effect on masticatory muscle function as measured by EMG. This approach allowed the inclusion of diverse experimental approaches in clinical and pre-clinical studies to thoroughly examine the potential relationship between psychological stress and masticatory muscle function, followed by the generation of specific hypotheses for future research.

## 2. Methods

The scoping review was conducted in accordance with the Joanna Briggs Institute (JBI) methodology for scoping reviews. It utilized the Preferred Reporting Items for Systematic Reviews and Meta-Analyses extension for scoping reviews (PRISMA-ScR) reporting guideline and checklist.

### 2.1 Protocol and registration

The methods of this review were registered on the Open Science Framework before the start of the study (Registration DOI: 10.17605/OSF.IO/Y7EBA).

### 2.2 Eligibility criteria

Studies were screened for eligibility via the PEO (Population, Exposure, Outcome) framework:

• Population: participants with or without TMD. Studies were excluded if the population of interest had specific comorbidities associated with higher pathological muscle tension (*e.g.*, dystonia) or disorders that decrease muscle tension or function (*e.g.*, stroke).

• Exposure: psychological stress (clinical or experimental).

• Outcomes: masticatory muscle EMG recordings.

Pre-clinical and clinical studies written in English or Dutch were included. Studies were excluded when the study design was a review or a case report. There were no limitations on publication year.

### 2.3 Information sources

The electronic databases searched included CINAHL, as well as EMBASE, Medline, and PsycINFO through the OVID platform. The search was performed in March 2025. A grey literature search was also undertaken through the following grey-literature-specific databases: BASE, OpenGrey, GreyMatters, Core, and Google Scholar. The reference list of all included sources of evidence were screened for additional studies via an Artificial Intelligence (AI) assisted methodology in Google Gemini (Gemini 1.5 Pro; Google, Mountain View, CA, USA), given its evidenced robustness in screening for relevant studies [17].

### 2.4 Search

The search strategy was built based on the keywords from the PEO research question in collaboration between the researchers and an Information Science Librarian. Keywords included “masticatory muscles”, “psychological stress”, and “electromyography”. The full search for Ovid MEDLINE is provided in **Supplementary material 1**.

### 2.5 Selection of sources of evidence

All retrieved studies were uploaded to Rayyan (https://www.rayyan.ai/, Rayyan Systems Inc., Cambridge, MA, USA), after which duplicates were identified by Rayyan and deleted by the research team. Abstracts were screened independently by two researchers (AW and HvdM) for inclusion using the above-mentioned eligibility criteria, followed by blinded full-text screening by the same researchers. A third researcher was available for reconciliation of any conflicts. The reference lists of all included studies were screened by an artificial intelligence tool (Google Gemini 1.5 Pro; Google, Mountain View, CA, USA) to undertake the snowballing search. The AI tool was provided with a description of the aims of the study and specific inclusion and exclusion criteria (see **Supplementary material 2** for prompts given to Google Gemini). To ensure this screening criterion was effective, a calibration exercise was undertaken whereby a preliminary search by AI of three papers was compared against a manual search. During this calibration exercise, the AI search was found to demonstrate good sensitivity but low specificity, resulting in a high rate of false positives (see **Supplementary material** for results), which required “human in the loop” screening. This is in keeping with recent evidence [17] demonstrating the potential for AI to expeditiously screen a reference list with coherent screening criteria with comparable sensitivity to human screeners.

### 2.6 Data items and charting process

Data from the included studies were extracted into a pre-defined template in Microsoft Excel, which was developed for this review. Initially, the following data were extracted: title of study, authors, publication date, country of study, study aims, study design, study setting, study population, model of stress exposure, muscles evaluated, muscle function during EMG, EMG outcome, and key study findings.

The extraction document underwent one minor revision after full-text screening had been completed. The amendment to the extraction process was the addition of a field to capture whether the studies included stated whether elevated stress was successfully achieved by the experimental stressor, *i.e.*, a manipulation check.

### 2.7 Critical appraisal

The JBI Critical Appraisal Checklist for Quasi-Experimental Studies was selected to assess the risk of bias across the included experimental studies that included a manipulation check, as it optimally aligns with their non-randomized, experimental methodologies [18]. The primary objective of these studies was to evaluate physiological reactivity, specifically muscle activity measured via EMG, in response to an active experimental manipulation, such as exposure to standardized laboratory stressors. Because participants were subjected to a controlled intervention with continuous or pre- and post-exposure measurements, these designs extend beyond a purely observational framework; hence the use of the JBI checklist for Quasi-Experimental Studies.

### 2.8 Data synthesis

Due to the explorative nature of this review and the expected heterogeneity of the included studies, there was a narrative data synthesis based on the type of study (pre-clinical *vs.* human), as well as the type of stress (experimental *vs.* observational). The experimental studies were then divided into those that had undertaken manipulation checks (self-reported, biological proxies, or other proxies) and those that had omitted a manipulation check.

## 3. Results

### 3.1 Selection of sources of evidence

The initial search found 167 titles. After removal of duplicates, 139 titles and abstracts were screened for eligibility, resulting in 55 papers included for full-text review. Thirty-four studies were subsequently included. A snowballing review of citations and references was then performed on the 34 included papers using the AI tool Google Gemini (Gemini 1.5 Pro; Google, Mountain View, CA, USA) in October 2025. From this process, 142 more titles were suggested, and title screening led to the exclusion of 122 articles, leaving 20 abstracts for review. After abstract review, ten full texts were reviewed, and four studies were deemed eligible and included in the final review. The grey literature search did not yield any studies beyond those already identified. See the PRISMA flow chart (Fig. 1).

**Fig. 1.** PRISMA flow chart of study selection.

### 3.2 Characteristics of sources of evidence

**Supplementary Table 1** (Ref. [19, 20, 21, 22, 23, 24, 25, 26, 27, 28, 29, 30, 31, 32, 33, 34, 35, 36, 37, 38, 39, 40, 41, 42, 43, 44, 45, 46, 47, 48, 49, 50, 51, 52, 53, 54, 55, 56]) demonstrates the results of included studies. Of the 38 included studies, nine were pre-clinical studies and 29 were human studies. Of the pre-clinical studies, three were experimental studies exploring masticatory muscle EMG after stress exposure, and the remaining three were experimental studies attempting to elucidate the biological mechanisms behind the masticatory muscle response to psychological stress. It was decided to keep these latter three studies for their interesting insights into putative physiological mechanisms underlying the results identified in the review. Three of the human studies were cross-sectional observational studies, whilst the remaining studies were experimental studies measuring masticatory muscle EMG after the participants were exposed to experimental stress. Of the 38 included studies, the largest contribution came from the USA (15/38). The oldest study included in the review was published in 1971, demonstrating that this topic has been researched for over fifty years. Most experimental studies (n = 20) explored EMG change when exposed to stress between a clinical population and a control population. The remaining nine studies explored the EMG activity of the muscles of mastication in an asymptomatic cohort. Of the clinical populations studied, all but three of the studies’ clinical samples had masticatory myalgia as a primary diagnosis. The remaining three clinical samples were patients with a broader topography of pain (fibromyalgia [32], myofascial pain of the craniomandibular region [35] and tension-type headache [34]), but the EMG analysis measured EMG activity in one of the muscles of mastication. The masseter muscle was the most studied muscle analysed (31/38) followed by the temporalis (20/38).

### 3.3 Critical appraisal within sources of evidence

Out of the twelve studies that performed a manipulation check, nine studies had a low risk of bias, one had a moderate risk of bias, and two had a high risk of bias. The bias mostly came from selection and allocation bias (three studies) and bias related to confounding factors (one unclear and two not applicable). No bias related to temporal precedence or to the assessment, detection, and measurement of the outcome was identified. The full assessment per study can be found in **Supplementary Table 2**.

### 3.4 Results of individual sources of evidence

The results of all individual studies are depicted in **Supplementary Table 1**. The three pre-clinical studies elicited stress by putting horses in social isolation [19] or exposing rodents to electric foot shock [20, 21]. The studies of Rosales [21] and Rankins [19] found no difference in stress biomarkers at the end of the test between stressed and non-stressed animals. All studies did, however, find elevated EMG levels of the masseter and/or temporalis muscles compared to the non-exposed groups.

There were three pre-clinical mechanistic studies performed in mice and rats that used a restraint stress model to prevent any physical movement, without pain, which induces stress in animals [22, 23, 24]. The study by Zhao [23] measured the presence of stress/anxiety through the “open field” and “elevated plus maze” test, which showed that there was stress-induced anxiety present in the studied mice. All three studies identified increased masseter muscle EMG activity.

Out of the three observational studies, there were two studies on students [25, 26] that assessed stress levels based on the Perceived Stress Scale (PSS). The third study included females with and without TMD, where the Trier Inventory for the Assessment of Chronic Stress (TICS) questionnaire was used [27]. All three studies showed an increase in masticatory muscle activity with an increase in stress levels, and the study of Schmitter *et al*. [27] also showed that women with TMD have higher EMG levels than those without TMD.

Finally, twenty-nine studies examined the influence of experimental stress on masticatory muscle activity in adults [28, 29, 30, 31, 32, 33, 34, 35, 36, 37, 38, 39, 40, 41, 42, 43, 44, 45, 46, 47, 48, 49, 50, 51, 52, 53, 54, 55, 56]. Out of these, twelve studies found that the experiment indeed increased perceived stress levels through either a subjective manipulation check or biological proxies of stress physiology. Seven studies used self-reported manipulation checks, and five used biological proxies. The other fourteen studies did not evaluate whether the experiment actually increased stress levels.

One study identified that awake bruxism events were elevated during stress tasks [28], whilst another did not find an increase in awake bruxism events even though it did find an increase in masticatory muscle activity [30]. Four studies did not find increased masticatory muscle activity during stress tasks [33, 37, 40, 43]. There were twenty-five studies that did find an increase in masticatory muscle activity during an experimental stress task [28, 29, 30, 31, 32, 34, 35, 36, 38, 39, 41, 44, 45, 46, 47, 48, 49, 50, 51, 52, 53, 54, 55, 56]. Sixteen of these studied people with TMD compared to controls [28, 29, 30, 32, 41, 42, 45, 46, 47, 48, 49, 50, 51, 52, 53, 54], and from these studies, eight studies reported a statistically increased level of masticatory muscle activity in patients with TMD compared to controls [29, 41, 48, 49, 50, 51, 52, 54], four studies showed no statistical difference in EMG response to stress between patients with TMD and controls [42, 45, 47, 53], in two studies this data and association were unclear [19, 21], in one study stress did not affect EMG in either group [40] and in one study the control group EMG response was greater than that observed in the TMD cohort [32]. The two studies that compared myogenous TMD to a temporomandibular joint disorder also did not find differences between the types of TMD [48, 53]. One study did not look at TMD but rather at the presence of bruxism [28], and found that people with bruxism have increased masticatory muscle activity during the stress test compared to non-bruxers.

### 3.5 Synthesis of results

Pre-clinical studies show that stress exposure increase masticatory muscle activity, both when stress levels are objectively increased and when they are not verified by manipulation checks. Clinical studies in humans show the same trend, where the majority of the studies show that increased stress levels, experimental or clinical, also increase masticatory muscle activity. This appears to be even more present in people with TMD. Fig. 2 is a summary of results.

**Fig. 2.** Summary of results that show that the connection between increased stress levels and increased masticatory muscle activity is present, but not necessary for stress experience to increase the masticatory muscle activity in animals or humans. TMD: Temporomandibular disorders.

## 4. Discussion

The purpose of this scoping review was to identify, examine, and map the available literature exploring the relationship between psychological stress and masticatory muscle EMG. The identified literature included both pre-clinical animal studies and clinical human studies. There was also a difference between experimental stress, or regular “clinical” stress, within the included studies. The key findings are that there is a connection between increased stress levels and increased masticatory muscle activity, but a stress experience is not necessary for an increase in masticatory muscle activity in animals or humans.

### 4.1 Findings from pre-clinical studies

All six pre-clinical studies reported elevation of masticatory muscle EMG when the animals were exposed to a stressor [19, 20, 21, 22, 23, 24]. Five of the six studies were delivered to rodents [20, 21, 22, 23, 24], and one study was conducted on horses [19]. These results allude to a consistent response in masticatory muscle EMG by mammals when they are exposed to a stimulus deemed as psychologically stressful. Due to their non-verbal status, their behaviours and biomarkers are used as proxies of their emotional state. Of the three pre-clinical studies evaluating masticatory muscle response to stress, only one of the studies [19] used cortisol levels to determine whether stress response had been elicited. Consequently, conclusions that stress was successfully induced in the remaining studies should be interpreted cautiously.

### 4.2 Findings from pre-clinical mechanistic studies

Three pre-clinical studies aimed to explore potential biological mechanisms that underpin the apparent causal relationship between stress and masticatory EMG in animals [22, 23, 24]. One study explored the activity of the locus coeruleus (LC) in mice under chronic restraint stress [22]. The LC is situated in the pons in both humans and mice, and has projections to the mesencephalic trigeminal nucleus (Vme) and can modulate the excitability of the Vme. The included study [22] demonstrated elevated activity of the LC and masticatory muscle EMG in their “stressed rats”, suggesting the LC may regulate this relationship by its involvement in sensitization and fear potentiation.

Another study explored the activity of the central amygdala and its projections to the Vme [23]. The amygdala is also key in humans for eliciting the physiological stress response and has projections to the LC with associated bidirectional feedback loops between the two structures [57]. They demonstrated increased masticatory muscle EMG concurrently with elevated amygdala activity in their rat stress model. Finally, the third study demonstrated an association between masticatory muscle tone and the presence of glutamate and glutaminase in the trigeminal motor nucleus [24]. Glutamate is an abundant excitatory neurotransmitter found in the LC and may be the main amino acid that mediates the relationship between stress, the amygdala, and oromotor tone. Notwithstanding the aforementioned limitations of pre-clinical studies, evidence is beginning to elucidate the mediating neurobiology & biochemistry that may explain the relationship between stress and masticatory muscle tone.

Caution must be applied when extrapolating from animal studies to humans; however, pre-clinical studies remain a valid tool to undertake early research in emerging fields of knowledge or in scientific inquiries that are impracticable in humans [58]. The presumption that animals share a similar emotional spectrum to humans remains controversial and is currently a sphere of scientific enquiry [59]. As such, even though these studies may give some insights into the biology that may underpin oromotor activity in response to psychological stress, they will most likely never fully explain the mechanisms that may be salient in humans.

### 4.3 Observational studies

Three studies used an observational cohort methodology, meaning there was no experimental stress but only clinical day-to-day stress [25, 26, 27]. Even though all three studies looked at masticatory muscle activity, they each had a different timing of EMG measurement, as well as different methods of measuring EMG levels, making it challenging to compare the results of these studies. One study assessed masticatory muscle EMG at rest [26], one evaluated Maximal Voluntary Contraction (MVC) EMG [25], and the final study measured masticatory muscle EMG as a means to record sleep bruxism episodes at night [27]. Despite these differences in EMG measurements, there is a trend for a positive association between increased stress levels and increased masticatory muscle activity amongst the included studies. However, one study used the PSS-14 scale as a measure of stress [26], which showed only a small mean change over time that may not be possibly be clinically relevant. Unfortunately, the responsiveness of the PSS-14 is unknown. Furthermore, although observational cohort studies offer opportunities to understand trends and associations in real-world populations, they cannot reliably establish causal relationships due to the inability to control confounding factors. As such, they may not represent as the most robust study design for exploring the relationship between masticatory muscle EMG and psychological stress.

### 4.4 Experimental studies

A large variety of experimental methodologies were used to elicit psychological stress in the study participants. From the twenty-nine experimental studies, there were sixteen different methodologies used. The most commonly used methodologies were mental arithmetic tests [39, 42, 45, 47, 51] and computer-based speed tests [34, 35, 36, 40, 43], both of which were used in five studies each. Twenty-five of the twenty-nine experimental studies reported a statistically significant increase in masticatory muscle EMG after stress exposure within their experiments. Of the four studies that did not demonstrate an increase in masticatory muscle EMG, two did not monitor stress elicitation [33, 37], and the remaining two studies [40, 43] measured changes in “perceived tension”. Therefore, it cannot be established for certain that within these four studies, stress was present, which could explain why there was no increase in masticatory muscle EMG. “Perceived tension” can serve as an index of psychological stress; however, it may better serve as a reflection of the physical sequelae of stress induction rather than a direct report of the presence of psychological stress, and as such may be a less robust psychometric measure of the state of psychological stress. Notably, contemporary research indicates that the effectiveness of stress elicitation protocols is highly variable, with specific tests targeting different branches of stress physiology [60]. Consequently, studies that failed to observe a change in EMG activity may have utilised protocols poorly suited for eliciting a neuromuscular response; in such cases, the null result may reflect the limitations of the stressor rather than a lack of physiological effect.

The results reported from the studies that did find a stress response suggest psychological stress may have a reliable effect on elevating masticatory muscle EMG. However, caution is warranted here given the inconsistent presence of manipulation checks for stress exposure, thereby making it difficult to definitively confirm the observed changes in EMG were as a direct result of psychological stress elicitation.

### 4.5 Experimental studies with manipulation checks

Fifteen of the twenty-nine studies assessed whether psychological stress was successfully elicited through a manipulation check. Of these fifteen studies, three different methodologies were used. Five of these fifteen studies used biological proxies of stress physiology to measure if a stress response was elicited (Heart Rate (HR) & Blood Pressure (BP) [32], Heart Rate Variability (HRV) [38], HR [42, 45], HR & BP & Skin conductance [47]). Seven studies evaluated if psychological stress was induced via a subjective 11-point Likert scale [29, 30, 31, 36, 39, 40, 41, 51], one of which used the Emotional Assessment Scale to discern a stress response [44]. Such measures have recognised construct validity to provide a manipulation check for psychological stress [61]. The remaining three studies measured the participants’ change in “perceived tension” to the experimental stressor as a manipulation check via a 10-point Likert scale. Of the studies that did undertake a manipulation check via direct subjective assessment of stress elicitation or objective physiological proxies, the authors reported that their stress induction paradigm successfully increased participants’ levels of stress. Despite the need for caution in interpreting the results of the twenty-nine experimental studies, it is noteworthy that, of the twelve studies that undertook a manipulation check and reported an increase in masticatory muscle EMG, nine studies demonstrated low risk of bias following critical appraisal. Consequently, some confidence in the results of this subgroup of studies is warranted.

### 4.6 Experimental studies without manipulation checks

Of the fourteen studies that did not undertake a manipulation check, six employed stress elicitation strategies that had also been used in experiments demonstrating successful stress induction in the studies with manipulation checks. While this provides some indirect support for the likelihood of stress induction in these six studies, it cannot constitute definitive verification of stress induction. Additionally, while fourteen studies had not delivered a manipulation check, it cannot be assumed that their results are less valid despite the absence of internal verification of the stress induction paradigm; rather, greater caution should be applied when considering their findings.

### 4.7 Clinical implications

The majority of the studies in this scoping review reported that psychological stress can increase masticatory muscle EMG. Furthermore, a systematic review by Szyszka-Sommerfeld *et al*. [7] investigating the relationship between masticatory muscle EMG and masticatory myalgia has demonstrated a high comorbidity between these factors such that elevated masticatory muscle EMG may be pathognomonic of masticatory muscle myalgia. Drawing from our review’s findings and the results from the Szyszka-Sommerfeld *et al*. [7] systematic review, it would be reasonable to suspect psychological stress as an antecedent of developing painful myogenous TMD or as a driver of ongoing myogenous TMD. Consequently, there is a compelling argument that psychological stress should be routinely investigated in patients with myogenous TMD and, if present, then addressed through suitable means. It should be noted that behavioural science contends that the experience of psychological stress is highly individualistic, although biological responses are considered generally universal and widespread throughout human physiology. A comprehensive framework illustrating the diverse physiological consequences can be found in the review by Shchaslyvyi *et al*. [62]. They articulate that multiple systems within the human body are mobilised by psychological stress, even at the genomic level, where stress can modulate DNA transcription and protein synthesis.

Despite some ambiguity in the data collected herein, there is a suggestion that EMG amplitude is greater in those with TMD. The mechanisms underlying this are speculative but may reflect that individual who develop TMD represent a phenotype with underlying premorbid “priming” of their stress-motor pathways. Alternatively, the presence of myalgic pain may in itself create a state of neuromuscular hyper-reactivity, thereby increasing stress-induced muscle activity. Additionally, one cannot ignore the notion that pain is, in its own right, an aversive experience that may compound these aforementioned issues by inducing psychological stress, thereby contributing to a “vicious-cycle phenomenon”.

Changes in Masticatory Muscle EMG are unlikely to be the sole reason for the development of masticatory muscle pain. The relationship between stress and pain is a relatively recent inquiry, and as such, comprehensive models for their relationship remain an ongoing research quest. That said, established research has shown that stress impacts various pain-modulating systems in such a way as to induce sensitisation of the pain neuromatrix [63]. Endocrine, immune, endocannabinoid, neurotransmitter, and pain neuromatrix morphology have all been shown to maladapt to chronic stress exposure [64]. Therefore, combined with the neuromuscular responses to stress we have found within our review, it can be argued that stress constitutes a dynamic risk factor to the development and exacerbation of painful myogenous temporomandibular disorders. Furthermore, although stress is not a standalone psychological disorder within the Diagnostic and Statistical Manual of Mental Disorders (DSM-5-TR), evidence suggests deleterious levels of stress are more prevalent than clinical diagnoses such as anxiety and depression [65]. Consequently, psychological stress may present a more significant societal health burden than many formally recognised psychopathologies.

### 4.8 Limitations of the study

Several limitations to this study must be acknowledged. Although scoping reviews offer the opportunity to map and explore the breadth and depth of a topic, they typically lack formal quality appraisal of the studies they identify. In this study, an attempt to address this limitation was undertaken, but only in a focused manner, and as such, several other studies remain unappraised. Furthermore, the heterogeneity of stress elicitation methods was significant, making it difficult to compare the findings with one another to form a single cohesive conclusion. Nonetheless, the variety of apparently successful stress modalities used offers insight into the notion that it can be a by-product of varied stimuli and therefore it should be respected as a dynamic entity. Most included studies were laboratory-based, typically capturing physiological responses to the stress exposure in the short-term, leaving a paucity of understanding regarding the long-term physiological consequences of prolonged stress exposure. Finally, despite attempts to ensure a comprehensive literature search was undertaken, some studies, notably non-English or Dutch language studies, may have been missed.

## 5. Conclusion

This scoping review has highlighted and mapped the significant body of research exploring the relationship between psychological stress and masticatory muscle activity, as quantified by electromyography. Our broad synthesis of the evidence suggests that psychological stress may reliably increase masticatory muscle EMG; however, caution is warranted given the paucity of manipulation checks within the included studies, and masticatory myalgia is likely a complex and multi-faceted phenomenon. Pre-clinical research is beginning to elucidate the potential underlying mechanisms of this relationship; however, generalisability to humans must be approached cautiously. Although muscle overloading is likely a consistent contributory factor, it is likely not the sole mediator of myalgic pain. That said, the findings from this scoping review warrant that psychological stress be consistently evaluated as part of a dynamic biopsychosocial assessment of patients with myogenous TMD.

## Supplementary Material

Supplementary material associated with this article can be found, in the online version, at https://files.jofph.com/files/article/2075415538954059776/attachment/Supplementary%20material.docx.


