# Supplementary material for: The effect of psychological stress on masticatory muscle electromyography—a scoping review
Source: J Oral Facial Pain Headache. 2026 Jul 12;40(4):9–18. doi: 10.22514/jofph.2026.047 (PMC13434148; doi:10.22514/jofph.2026.047)
Supplement: Supplementary file 1 [file Supplementary-material.docx]

Supplementary material

# Supplementary material 1: Search strategy.

The following search strategy was used to screen for possible articles:

Databases searched were Medline, Embase and CINAHL with no date or language restriction.

Ovid MEDLINE(R) ALL “1946 to 04 March 2025”

(1). exp Masticatory Muscles/	14,097

(2). (masticatory muscle* or masseter or temporalis).ti. or (masticatory muscle* or masseter or temporalis).ab. 13,328

(3). 1 or 2	20,221

(4). exp Stress, Psychological/	163,828

(5). (psychological stress* or mental stress* or anxiety or psychological distress).ti. or (psychological stress* or mental stress* or anxiety or psychological distress).ab. 332,816

(6). 4 or 5	459,807

(7). exp Electromyography/	87,455

(8). (EMG adj3 measur*).ti. or (EMG adj3 measur*).ab. 2534

(9). (electromyography or EMG).ti. or (electromyography or EMG).ab. 57,139

(10). 7 or 8 or 9	107,217

(11). 3 and 6 and 10 121

Please acknowledge this search in any papers/presentation as:

Literature Search: Effect of psychological stress on masticatory muscle EMG (Nottingham University Hospitals Library Service).

# Supplementary material 2

Artificial Intelligence screening prompt:

I am undertaking a scoping review to explore whether psychological stress affects masticatory muscle electromyography (EMG). I have 34 papers included and I would like you to screen their references for any potential papers to be included. My inclusion criteria is: human (clinical or non-clinical populations) or animal studies evaluating the effect of psychological stress induction (either by experimental means or clinical) on masticatory muscle EMG. Exclusion criteria are: Reviews and case reports, populations with neurological disease such as stroke or dystonia.

Supplementary Table 1. Characteristics and findings of individual studies stratified by pre-clinical and clinical studies.

| Reference | Author/Year/Country | Study aims & MOM Muscles tested | Sample Mean Age (yr) | Model of stress exposure | Success of stress exposure (Y/N) | Study findings |
| --- | --- | --- | --- | --- | --- | --- |
| Pre-clinical studies | | | | | | |
| [19] | Rankins *et al.* 2022 USA | To determine the effect of stress on muscle activity  Masseters | 8 horses (4 M/4 F) 13 yrs | Sham clipping & social isolation stress tests | (Y) | Masseter EMG elevated |
| [20] | Wu *et al.* 2013 China | Observe the effect of psychological stress on MOM  Masseters & Temporalis | 120 Sprague-Dawley rats  (?) | Electric foot shock exposure | (?) | Masseter & Temporalis EMG elevated |
| [21] | Rosales *et al.* 2002 Japan | To assess bruxism behaviour as a response to stress exposure  Masseter | 10 Wister rats  (9 weeks old) | Electric foot shock exposure | (N) | Masseter EMG elevated |
| Pre-clinical mechanistic studies | | | | | | |
| [22] | Liu *et al.* 2024 China | Examine the role of locus coeruleus neurons in masseter overactivity induced by stress | 16 Mice  (?) | Restraint stress model | (?) | Masseter EMG elevated & locus coeruleus neurons mapped |
| [23] | Zhao *et al.* 2024 China | To map projections from Amygdala to trigeminal nucleus during stress  Masseter | Mice  (8–10 weeks old) | Restraint stress model | (Y) | Masseter EMG elevated  GABAergic projections from Amygdala to TCN mediate this relationship |
| [24] | Song *et al.* 2014 China | Determine the mechanism of  the stress-induced increase in masseter muscle tone & the effect of lamotrigine on this | Eighty male Sprague–Dawley rats  (8 weeks old) | Restraint stress model | (?) | Masseter EMG elevated  This may be mediated by glutamate & glutaminase |
| Observational studies | | | | | | |
| [25] | Paz-Cortés *et al.* 2024 Spain | Evaluate the influence of stress on “TMJ status”  Masseter | 70 students  (21.75) | Academic year pre & post exams | (Y) | Masseter EMG increased as mean levels of stress increased |
| [26] | Owczarek *et al.* 2020 Poland | Assess “psychoemotional state” relative to master EMG  Masseter | 113 students  (21.8) | PSS-10 score taken relative to MOM EMG | (Y) | Elevated master EMG was found in those with higher PSS-10 scores |
| [27] | Schmitter *et al.* 2019 Germany | Assess stress and temporalis muscle activity in the form of sleep bruxism episodes  Temporalis | 44 females  (45.1)  22 with TMD & 22 controls | Four nights of EMG recording compared to psychosocial based questionnaire | (Y) | Sleep bruxism episodes measured by temporalis EMG were more frequent in those with higher stress levels and higher again in those with TMD |
| Clinical studies | | | | | | |
| [28] | Chattrattrai *et al.* 2023 USA | Investigate relationship between sleep & awake bruxism & stress  Masseter & Temporalis | 122 patients with TMD (37.9) & 46 controls (35.7) | 4-part stress test: Cold pressor test, mental arithmetic test, speech stressor & reaction time test | (?) | Awake bruxism events elevated during stress tasks |
| [29] | Janal *et al.* 2021 USA | Assess MOM EMG when exposed to stressor  Masseter & Temporalis | 124 female TMD patients (40.3) & 46 age matched controls | 4-part stress test: Cold pressor test, mental arithmetic test, speech stressor & reaction time test | (Y) | Stressor tests increased Masseter & Temporalis EMG, more so in TMD patients than in controls |
| [30] | Imbriglio *et al.* 2020 Canada | Evaluate if guided music listening intervention modulates MOM EMG & AB events  Masseter & Temporalis | 14 female TMD & 15 control subjects  (30.25) | 15-minute blocks of music; Pink stressful, relaxing & favourite noise/music | (Y) | Stressful music block increased MOM EMG but not AB events, unclear if this differed between groups |
| [31] | Cioffi *et al.* 2018 Canada | Evaluate if guided music listening intervention modulates MOM  Masseter | 21 healthy volunteers  (22) | 20-minute blocks of music: favourite, dissonant (stress) & relaxing | (Y) | Dissonant/stressful music increases Masseter EMG |
| [32] | Woda *et al.* 2013 France | Assess MOM EMG to stress stimulus in 3 different cohorts  Masseter & Temporalis | 30 TMD (37.64)  31 FMS (47.36)  32 controls (36.34) | The Trier Social Stress Test (TSST) | (Y)  No subjective assessment but HR & BP measured | TSST increased MOM EMG in all 3 groups but the most in controls and least in FMS group |
| [33] | Tanosoto *et al.* 2012 Japan | Assess “jaw-muscle activities” in response to acute experimental stress  Masseter | 11 healthy men (25.2) & 5 healthy females (23.0) | Modified Paced Auditory Serial Addition Task test (PASAT) | (?) | PASAT test did not affect Masseter EMG |
| [34] | Leistad *et al.* 2005 Norway | Evaluate muscle activation during & after stress  Temporalis | 44 controls (39.7)  40 headache patients (22 migraine & 18 TTHA) (36.8) | Computer based “grid placing test” | (?) | Temporalis EMG increased only in the Migraine patients’ group |
| [35] | Nilsen *et al.* 2005 Norway | Study the effect of stress on FMS *vs.* chronic shoulder/neck pain *vs.* controls neck & MOM EMG  Temporalis | 35 Controls (39.7)  23 FMS (48.3)  29 SNP (41.1) | Two-choice reaction-time test on a monitor, lasting one hour | (?) | Temporalis EMG increased in the SNP group only during & post stress test & post stress test for FMS group |
| [36] | Bakke *et al.* 2004 Denmark | Assess saliva cortisol & flow rate & MOM EMG in response to minor stress  Masseter & Temporalis | 23 women  (43.5) | Two fast paced computer tasks | (Y) | Masseter & Temporalis EMG increased due to the stress test |
| [37] | Hidaka *et al.* 2004 Japan | Explore relationship between mental stress & EMG of Masseter  Masseter | 10 healthy females  (25.3) | 30 minutes  Chinese character identification test | (?) | Masseter EMG did not change during the “Chinese character identification test” |
| [38] | Hidaka *et al.* 2004 Japan | Assess the ANS & EMG response to a “long mental task”  Masseter & Temporalis | 20 healthy females  (25.2) | 120 minutes  Chinese character identification test | (Y)  No subjective assessment but HRV recorded | Both Masseter & Temporalis EMG increased although Temporalis increased more than Masseter |
| [39] | Tsai *et al.* 2002 Taiwan | To study the effect of stress on MOM  Masseter & Temporalis | 35 healthy dental students  (20–34) | Mental arithmetic task | (Y) | Masseter & Temporalis EMG elevated |
| [40] | Bansevicius *et al.* 2001 Norway | Assess EMG changes in MOM of FMS patients under stress  Temporalis | 15 FMS (47.5) & 15 group-matched controls (46.9) | Two-choice computer based reaction time test | (Y)  Subjective “tension” not stress assessed | Temporalis EMG did not change as a result of the stress test in either group |
| [41] | Nicholson *et al.* 2000 USA | Evaluate influence of stress test on masseter EMG in TMD patients & controls  Masseter | 25 TMD patients (34.9) & 25 controls (30.4) | Four-phase stress experiment that ran over 90 minutes | (?) | Masseter EMG increased as a response to the stressor and did more so in the TMD group |
| [42] | Carlson *et al.* 1998 USA | To identify psychophysiological variable of those with TMD & controls  Masseter & Temporalis | 35 TMD patients (30.17) & matched controls (30.14) | Mental arithmetic test | (Y)  No subjective assessment but HR was measured | Masseter & Temporalis EMG elevated as response to stressor but no between group difference |
| [43] | Bansevicius *et al.* 1997 Norway | Evaluate facial & neck muscle EMG from long duration mental stress  Temporalis | 36 pain-free subjects  (34.6) | Two choice reaction time test at a computer for 60 minutes | (Y)  Subjective “tension” not stress assessed | No change in temporalis muscle EMG in response to stressor test |
| [44] | Ruf *et al.* 1997 Norway | Assess Masseter EMG whilst chewing &maximal bite during a stressful exam  Temporalis & Masseter | 15 pain free dental students  (23) | Stressor was their exam | (N)  “perceived helplessness” assessed | MOM EMG was elevated during the exam period/stress exposure |
| [45] | Curran *et al.* 1996 USA | Explored psychophysiological factors between TMD patients & controls  Temporalis & Masseter | 23 TMD & 23 age matched controls  (23.9) | Mental arithmetic test | (Y) | Both Masseter & Temporalis EMG increased as a response to the stressor test but there was no difference between the groups |
| [46] | Intrieri *et al.* 1994 USA | To explore CV & Masseter muscle EMG under stress in two groups  Masseter | 10 women with TMD (33) & 12 female controls (32.58) | Shock avoidance stressor test | (Y)  Anxiety rather than stress subjective but  HR was recoded | Both groups responded with increased Masseter EMG with no between group difference |
| [47] | Carlson *et al.* 1993 USA | Evaluate possible psychological & physiological differences between TMD patients & controls  Masseter | 34 TMD patients (34.1) & 18 controls (−) | Mental arithmetic test | (Y)  HR, BP & Skin conductance recorded | Both groups responded with increased Masseter EMG with no between group difference |
| [48] | Flor *et al.* 1991 Germany | Assess EMG response to stress in 4 different groups of patients  Masseter | 10 MTMD  12 TMJD  12 CLBP  12 controls  (36.2) | Participants were to recall a stressful event in their lives | (Y)  Subjective “tension” not stress assessed | Masseter EMG elevated in both TMD groups but not in CLBP & control group  No difference between TMD groups |
| [49] | Montgomery *et al.* 1990 USA | To explore the effect of short- & long-term stress exposure on masticatory muscle EMG in TMD & control subjects  Masseter | 9 TMD subjects & 22 controls  (38) | A reaction time task | (?) | The reaction time task elevated Masseter EMG in both groups but in the TMD group more |
| [50] | Kapel *et al.* 1989 USA | To test if those with MTMD display increased facial muscle activity under stress  Masseter & Temporalis | 20 MTMD patients & 20 controls  (?) | Participants watched “idyllic” & “horrific” (stressor) film clips | (?) | The stressor elicited elevated masticatory muscle EMG, more so in the MTMD group |
| [51] | Katz *et al.* 1989 USA | To test the effect of experimental stress on masticatory muscle EMG in those with TMD & controls  Masseter & Temporalis | 20 TMD (31.2) & 20 age-matched controls | Numerical memory test | (Y) | The stressor elicited elevated masticatory muscle EMG, more so in the TMD group |
| [52] | Dahlstrom *et al.* 1985 USA | To test the effect of experimental stress on masticatory muscle EMG in those with TMD & controls  Masseter | 20 TMD (32.0) & 20 age-matched controls | Needle threading test followed by puzzle making test | (?) | The stressor elicited elevated masticatory muscle EMG, more so in the TMD group |
| [53] | Moss *et al.* 1984 USA | To test the effect of experimental stress on masticatory muscle EMG in two TMD groups & controls  Masseter | 10 TMD (29.5), 10 with TMJ sounds & no pain (28.4) & Control (25.8) | 4-part stressor test:  startle stimulus, buzzer series, serial arithmetic test, & pain threshold/tolerance tests | (?) | Masseter EMG was elevated as a response to the stress test however there was no between group difference |
| [54] | Mercuri *et al.* 1979 USA | To test the effect of experimental stress on masticatory muscle EMG in those with TMD & controls  Masseter | 20 TMD patients (30) & 20 age-matched controls | Card sorting exercise developed to induce stress | (?) | The stressor elicited elevated masticatory muscle EMG, more so in the TMD group |
| [55] | Rao *et al.* 1979 USA | To evaluate masseter EMG response between diurnal bruxists & non-bruxists  Masseter | 8 female bruxists & 8 female non-bruxists | Modified Stroop Test | (?) | Both groups responded with elevated Masseter EMG but bruxists response was stronger |
| [56] | Yemm *et al.* 1971 UK | To evaluate effect of stress on masticatory EMG  Masseter & Temporalis | 15 asymptomatic individuals | Flashing light reaction tests | (?) | Masseter & Temporalis EMG increased as a response to the stress test |

Key: Y: yes; N: No; ?: not evaluated; MOM: Muscle of Mastication; AB: Awake Bruxism; HR: Heart Rate; TTHA: Tension Type Headache; SNP: Shoulder Neck Pain; ANS: Autonomic Nervous System; CV: Cardiovascular; MTMD: Myogenous Temporomandibular Disorder; TMJD: Temporomandibular Joint Disorder; CLBP: Chronic Lower Back Pain; M: Male; F: Female; EMG: electromyography; TMD: Temporomandibular disorders; TSST: Trier Social Stress Test; PASAT: Paced Auditory Serial Addition Task; TCN: Trigeminocervical nucleus; PSS-10: Perceived Stress Scale-10; FMS: Fibromyalgia Syndrome; HRV: Heart Rate Variability; BP: Blood Pressure.

Supplementary Table 2. Results of critical appraisal.

| Author, year | 1. Bias related to temporal precedence | 2. Bias related to selection and allocation | 3. Bias related to confounding factors | 4. Bias related to administration of intervention/exposure | Bias related to assessment, detection and measurement of the outcome | | | 8. Bias related to participant retention | 9. Statistical Conclusion Validity | Risk of Bias |
| --- | --- | --- | --- | --- | --- | --- | --- | --- | --- | --- |
|  |  |  |  |  | 5. Multiple assessments? | 6. Similar outcome measurements? | 7. Reliable outcome measurements? |  |  |  |
| Janal, 2021 | Yes | Yes | Yes | Yes | Yes | Yes | Yes | n/a | Yes | LOW |
| Imbriglio, 2020 | Yes | No | n/a | n/a | Yes | Yes | Yes | n/a | Yes | HIGH |
| Cioffi, 2018 | Yes | Yes | Yes | Yes | Yes | Yes | Yes | n/a | Yes | LOW |
| Woda, 2013 | Yes | Yes | Unclear | Yes | Yes | Yes | Yes | n/a | Yes | LOW |
| Bakke, 2004 | Yes | Yes | Yes | Yes | Yes | Yes | Yes | n/a | Yes | LOW |
| Hidaka, 2004 | Yes | No | n/a | n/a | Yes | Yes | Yes | n/a | Yes | HIGH |
| Tsai, 2002 | Yes | No | Yes | Yes | Yes | Yes | Yes | n/a | Yes | MOD |
| Carlson, 1998 | Yes | Yes | Yes | Yes | Yes | Yes | Yes | n/a | Yes | LOW |
| Curran, 1996 | Yes | Yes | Yes | Yes | Yes | Yes | Yes | n/a | Yes | LOW |
| Intrieri, 1994 | Yes | Yes | Yes | Yes | Yes | Yes | Yes | Yes | Yes | LOW |
| Carlson, 1993 | Yes | Yes | Yes | Yes | Yes | Yes | Yes | n/a | Yes | LOW |
| Katz, 1989 | Yes | Yes | Yes | Yes | Yes | Yes | Yes | n/a | Yes | LOW |

n/a: Not Applicable; MOD: moderate.
